# Impact of the COVID-19 pandemic on family carers of those with profound and multiple intellectual disabilities: perspectives from UK and Irish Non-Governmental Organisations

**DOI:** 10.1186/s12889-022-14560-4

**Published:** 2022-11-16

**Authors:** M. A. Linden, T. Forbes, M. Brown, L. Marsh, M. Truesdale, E. McCann, S. Todd, N. Hughes

**Affiliations:** 1 School of Nursing and Midwifery, Queen’s University Belfast, 97 Lisburn Road, Belfast, BT9 7BL Northern Ireland; 2 School of Health and Wellbeing, University of Glasgow, Glasgow, Scotland; 3 Division of Nursing at City, University of London, London, UK; 4 School of Care Sciences, University of South Wales, Caerleon, Wales; 5 Department of Sociological Studies, University of Sheffield, Sheffield, England

**Keywords:** Intellectual disability, Non-Governmental Organisations, COVID-19, Qualitative, Focus groups

## Abstract

**Background:**

Family carers of people with profound and multiple intellectual disabilities (PMID) experienced a reduction in healthcare services due to the COVID-19 pandemic. Many subsequently turned to Non-Governmental Organisations who worked to support families. However, little research has sought to capture the experiences of family carers or identify effective interventions which might support them. To address these concerns we explored the views of Non-Governmental sector workers across the UK and Ireland who supported families people with PMID during the COVID-19 pandemic. We also sought to explore their views on the characteristics of online support programmes for family carers.

**Methods:**

This study employed a qualitative design using focus groups with participants (*n* = 24) from five Non-Governmental Organisations across the UK and Ireland. A focus group guide included questions on challenges, supports, coping and resources which helped during lockdown restrictions. Focus groups were held online, were audio recorded and transcribed verbatim. The resulting transcripts were pseudonymised and subjected to thematic analysis.

**Findings:**

Four themes were identified (i) ‘mental and emotional health’, (ii) ‘they who shout the loudest’ (fighting for services), (iii) ‘lack of trust in statutory services’ and (iv) ‘creating an online support programme’. Mental and emotional health emerged as the most prominent theme and included three subthemes named as ‘isolation’, ‘fear of COVID-19’ and ‘the exhaustion of caring’.

**Conclusions:**

The COVID-19 pandemic has increased the vulnerability of family carers who were already experiencing difficulties in accessing services and supports for their families. While Non-Governmental Organisations have been a crucial lifeline there is urgent need to design services, including online support programmes, in partnership with family carers which adequately address their needs.

## Introduction

The coronavirus disease (COVID-19) spread across the globe in 2020 resulting in death and long-term health challenges. Subsequent lockdown restrictions resulted in the curtailment of many social freedoms and the removal or scaling down of health and social care services [1]. Families who relied on social care services to provide care and support experienced significant stress [2] and reduced mental health [3, 4] which may have long-term implications for their health.

Family carers provide significant and ongoing care for their family members with intellectual disabilities [5]. Profound and multiple intellectual disabilities (PMID) refers to people with a severe learning disability and cognitive impairment that significantly affects their ability to communicate and live independently [6]. Due to severity of impairment, the burden of care experienced by family carers of those with PMID might be considerable.

People with PMID often have severe, life-long impairments including reduced vision, hearing and mobility, with associated complex physical and mental health conditions [7, 8]. They are at greater risk of experiencing multiple and complex physical health needs, including epilepsy, coordination disorders, respiratory infections, pneumonia, dysphagia, gastroesophageal reflux disorder, helicobacter pylori, constipation and urinary incontinence [9–11] These conditions require on-going management by family carers and services to prevent deterioration and minimise the potential of complications such as chest infection and pneumonia [12]. Services required by people with PMID and intellectual disabilities (ID) prior to the pandemic included physiotherapy, speech and language therapy, personal assistants, day activity centres, respite care, special education, mental health and social care [13–16], which were essential for their continued health and well-being. With the removal of in-person treatment due to pandemic restrictions, UK and Dutch studies showed increases in referrals to psychiatric services for deterioration in the mental health of people with ID [14, 17]. The removal of in-person physiotherapy services to treat coordination disorders meant that treatment was delivered online with the rehabilitation burden placed on family carers [3]. Prior to the pandemic many of these services had experienced cuts, or offered limited service provision, leaving people with disabilities feeling their needs were not being met [18, 19].

Research has shown that people with ID and their family carers experienced significant strain during the pandemic. An international online survey of carers and support professionals (*n* = 3754) conducted in 12 countries between August and September 2020 showed that carers experienced high levels of stress and depression [1]. The strongest predictor of carer well-being were changes in mood of the person they cared for [1]. Similarly, a survey of 323 Spanish carers of people with ID also documented increased levels of stress which was attributed to an increase in their caring role due to service closures [13]. A qualitative study conducted with eight English parents of people with ID during the first lockdown period of 2020 identified themes of powerlessness, coping (establishing a routine, sacrifice as the norm), support (lack of services, appreciation of technology) and reduced well-being (vulnerability of their family member, relentless demands of caring) [20]. Interviews with 24 people with ID living in England revealed experiences around a lack of consideration in government planning, cuts to social care provision, removal of opportunities for social contact and lack of vaccine prioritisation [21]. While evidence existed to show that people with ID were at increased risk of morbidity and mortality from Covid-19 [22] the UK government did not originally assign them priority status for vaccination [21].

Non-Governmental Organisations (NGOs) provide ongoing care and support for families of individuals with disabilities. Among others, these include supported living, residential services, day care, vocational training, supported employment, respite care, leisure and advocacy [23]. With the removal of services and support structures during the COVID-19 pandemic, NGOs became even more vital with many services moving to online support platforms to provide essential services for families [24]. These included, art therapy [25], sport and fitness [26] mental health support [21], and the provision of technology to enable online interaction [15]. The use of online platforms and telephone support services were a lifeline for many, whilst offering both opportunities (e.g. remote access, availability, reduced travel) and challenges (e.g. access to technology, reliability of technology) (REMOVED FOR BLIND REVIEW). While in the past families may have expressed a preference for face to face support, the COVID-19 pandemic demonstrated that some services were effectively delivered online [27, 28]. For example, videoconferencing was thought to be a useful tool and source of support for carers in staying in touch with day services [20], while online training to provide mental health support was shown to improve carer well-being [19]. However, few researchers have yet developed an evidence-based online programme to meet the needs of family carers. Programmes which include the perspectives of family carers will more closely align with their needs and be better suited in meeting these. Co-production of such a programme is an approach that offers great promise through recognising that traditional notions of expertise are complemented by the lived experience of service users [29]. Co-production offers opportunity to bring together diverse perspectives of key stakeholders to truly collaborate on a topic that is of mutual benefit [29]. Researchers have successfully employed co-production to investigate diet and weight management in individuals with ID [30], to identify health priorities for families of South Asian children [31] and to create an intervention for carers of people with dementia [32]. In addition to gaining the perspectives of family carers in creating a programme to meet their needs, the views of NGOs as key stakeholders in the lives of carers are an additional source of information. Non-Governmental Organisation workers (NGOW) have played an important role in supporting families of people with intellectual disabilities and had regular contact with families during the COVID-19 pandemic [23]. In addition to continued provision of many of their individual programmes, NGOs advocated for the needs of persons with ID at government; translated COVID-19 guidance within supported living settings and adapted their services to take advantage of new technologies [23]. As a key source of support for family carers during the pandemic, NGOs had regular contact with carers through their online activities and via telephone [23]. They built on already established relationships with carers and formed close bonds with them. They are therefore well placed to provide insights into the challenges family carers experienced.

The COVID-19 pandemic has brought about changes to the lives of many, however, to date little research has focused on the experiences of family carers of people with PMID. The research presented here sought to capture the experiences of family carers of people with PMID during the COVID-19 pandemic. Further, the project sought, with the aid of NGOW and family carers to develop an online support programme which would ameliorate the impact of the pandemic on carers. We sought to address two research questions: 1. What do NGOW perceive to have been the particular impacts of the COVID-19 pandemic on family carers of people with PMID?; 2. What features of an online programme would be helpful in alleviating the impact of the COVID-19 pandemic on family carers?

## Methods

### Design

This manuscript describes the first phase of data collection in a larger study intended to explore the experiences of family carers of people with PMID and develop a programme to ameliorate the impact of the COVID-19 pandemic. These findings describe the views of NGOW who provided support for these families during the pandemic. Employing a qualitative design allowed us to explore the experiences of NGOW during the pandemic and gain their perspectives on how the pandemic affected their clients.

### Participants

Twenty-four participants took part in five focus groups [33] across the four countries of the UK and The Republic of Ireland. Five NGOs took part in total. Participants were recruited from NGOs who provided support to family carers and comprised 20 females and 4 male members of staff. Five members of staff were also self-advocates meaning that they were also carers of a family member with an intellectual disability. Three of the NGOs had a regional remit whilst two were national. The characteristics of study participants are shown in Table 1.

**Table 1:** Demographic details of study participants

Characteristics	Category	Number (%)
Gender	Male	4 (17)
	Female	20 (83)
Ethnicity	Caucasian	24 (100)
Age range	25–34	4 (17)
	35–44	5 (21)
	45–54	7 (29)
	55–64	7 (29)
	65 +	1 (4)
Years in NGO sector	1–5 years	8 (33)
	6–10 years	3 (12.5)
	11–15 years	2 (8)
	16–20 years	4 (17)
	21 + years	3 (12.5)
	Missing data	4 (17)
Country	England	3 (12.5)
	Scotland	7 (29)
	Northern Ireland	6 (25)
	Republic of Ireland	3 (12.5)
	Wales	5 (21)

### Data collection

Five focus groups were held between September and December 2021. Non-Governmental Organisations who provided care to families of individuals with intellectual disabilities were invited to take part. We sought the views of those with a client facing role and employed convenience sampling for the purpose of recruitment. Letters of invitation were emailed to eligible staff (those with a client facing remit) by a gatekeeper in their organisation, followed by a participant information sheet approximately one week later. Those who expressed an interest in participating in the study shared their details with the gatekeeper who arranged a suitable time for the focus group to take place. Focus groups were conducted using an online platform with discussions recorded for later transcription and analysis. Focus group discussions lasted between 60 and 90 min. The focus group guide is included in Table 2. Informed consent and basic demographic details were gathered by means of an online questionnaire.

**Table 2:** Focus group guide

1. Can you tell me a bit about yourself and the people you care for?
2. Can you tell me what the pandemic was like for you and your clients?
*Potential prompts: Can you tell me what life was like for you before, during and after the pandemic? Can you tell me a bit about how these changes made you feel?*
3. Can you tell me what the most challenging aspects of the pandemic was for you?
*Potential prompts: Can you tell me more about that? Can you give me an example of that?*
4. Can you tell me what helped you and your clients cope during the pandemic?
*Potential prompts: Can you tell me more about that? Can you give me an example of that? Have there been any particular resources that have helped?*
5. We are hoping to create a support programme to help your clients, what do you think such a programme should contain?
*Potential prompts: Why do you think this is? Can you explain more about this?*
6. Where there any programmes or websites that you found helped?
7. What would an ideal programme look like to you?
8. How do you think such a programme should be delivered?
*Potential prompts: Why do you think this is? Can you explain more about this?*
9. Are there any topics that we haven’t covered today or that you would like to talk about before we finish the interview?

### Ethical considerations

Ethical approval was granted by the Faculty of Medicine Health and Life Sciences ethics review board (Ref: MHLS 21_38) at the Queen’s University of Belfast. Participants were required to provide written consent prior to participation and were fully informed of their right to withdraw, the limits of confidentiality and data protection. All procedures were conducted in accordance with the Declaration of Helsinki.

### Data analysis

The six stage process of thematic analysis [34] was employed to capture important features of the data in relation to the research questions. Themes were inductively developed and represent major motifs across the data.

### Rigour

To ensure rigour, the research protocol was first reviewed by two independent researchers as part of our application for ethical approval. The same researcher (TF) conducted all of the focus groups with recordings transcribed verbatim to ensure accurate accounts of participants’ views were captured. The researcher was an experienced psychologist with over twenty-one years familiarity with qualitative methods. Credibility of the analysis was achieved through triangulation by two members of the research team with direct quotations presented below to demonstrate confirmability.

### Findings

**Fig. 1:**
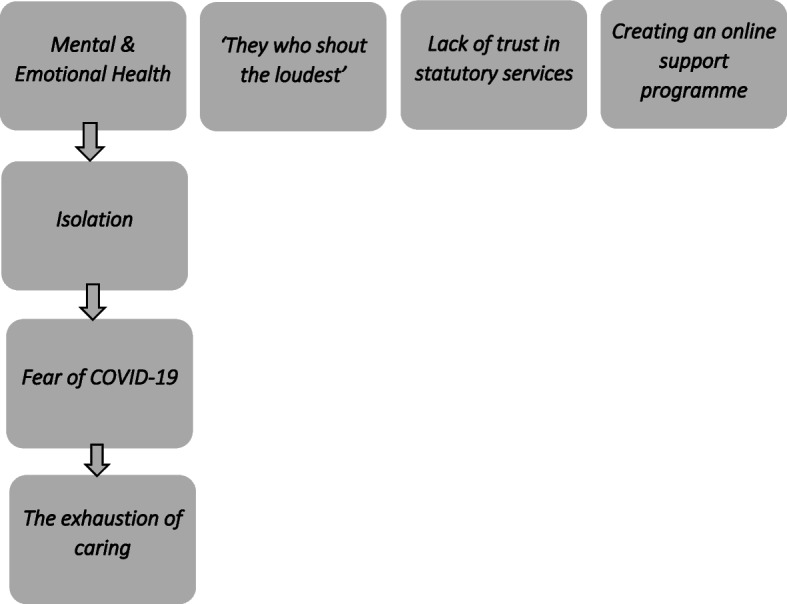
Summary diagram of themes

Thematic analysis resulted in four primary themes and three subthemes which are summarised in Fig. 1 and explained below.

#### Mental and emotional health

*I think families were scared and then confused and I think they have went through different stages of emotions since then, stages of grief and there has been different stages throughout the pandemic. There has been anger, frustration and a lot of negative emotion.* [Female, NGOW, 45-54 years old]

The pandemic was a highly emotive time for all and one of the main themes to emerge from the focus group interviews related to the impact of the pandemic on people’s mental health and emotional well-being; both family carers and the individuals they have been caring for, in addition to that of staff within the NGO sector. This theme was apparent in all of the focus groups and incorporated a broad range of ways in which people’s mental health had been impacted, for example, loneliness, fear, anxiety and uncertainty. These subthemes are presented in Sects. 1.1 – 1.4.

One participant commented on the impact of lockdown on their own mental health, and that of their team:

*I’ve really struggled with my mental health throughout lockdown like all of us have in different ways and there is not one of us that hasn’t been in tears in a meeting* [Female, NGOW, 45-54 years old]

Concern about the pandemic having a negative impact on mental health largely focused on that of family carers and those individuals they were caring for.

*We were really worried about people, some of our advocate team, we were seriously worried about what was happening to their mental health because of what was going on. I think mental health was definitely one of the worst things.* [Female, NGOW, 45-54 years old]

A participant in another focus group reflected on how successive events in family carers lives meant that there was a cumulative negative effect on their mental health:

*I think you could also look at all of our family carers as having gone through multiple experiences of trauma because it’s just been horrendous* [Female, NGOW, 55-64 years old]

However, there was an element of hope from some of the NGOW who felt that, while they had gone through difficult times, they also felt supported by their colleagues and were thankful for them.

*We have all cried, self-advocates have cried and we have had carers or family members in tears and it’s trying to just say we are all going to get there, you are not alone, we are all in this together, which is great for our team.* [Female, NGOW, 45-54 years old]

Concern for the long-term impact of the pandemic on family carers’ mental health and that of their family members was evident:

*These people need the help, these people are in crisis and I feel like I have been alongside my friends and they are on their knees. They are coping, some of them aren’t coping and it’s really, really tough and there doesn’t seem to be a light at the end of the tunnel and the support still isn’t coming.* [Female, self-advocate, 35-44 years old]

Non-Governmental Organisation Workers, who were also family carers or self-advocates, described the impact of lockdown restrictions on the mental health of family members with learning disabilities. This was often discussed to the exclusion of the impact of the pandemic on their own mental health. One self-advocate said:

*I think for me I suppose it reminded me we talk through our anxieties, our losses and our despair at lockdowns and what is happening. She was quietly experiencing all of those emotions, and that loss and not saying very much but it was very real.* [Female, self-advocate, 55-64 years old]

Participants highlighted that while some people with PMID may not be able to fully communicate their needs it did not mean that they had not been emotionally and psychologically impacted by the pandemic. Some self-advocates described how their family members’ behaviour had changed as a result of stress brought on by lockdown restrictions. For example:

*He [my son] was actually showing a lot of signs of stress by then… …[My son] was an extremely easy going guy, never had any behavioural issues with him in the sense of being aggressive or anything like that. If the staff took him out in the car just to have his hour out or they took him out in the wheelchair for an hour, when they came back and they were trying to get him back indoors he was kicking them, he was punching, he was trying to close the car door. There were all sorts of reactions that were saying he didn’t want to go back in, he was happy being out-and-about and that was so unlike him.* [Female, self-advocate, 65+ years old]

#### Isolation

Isolation is often an issue for families of people with more severe intellectual disabilities or PMID, with the pandemic exacerbating this. Families had many of the services and supports they regularly relied upon removed, leaving them feeling powerless and alone:

*It’s an isolated group that we work with anyway but when services stopped for them a lot of folk withdrew their children before the schools finished and services began to stop.* [Female, NGOW, 45-54 years old]

*It’s like we said they are very isolated and particularly when they have young children, normally parents meet at activity clubs and school gates and our families don’t have that. Also with family carers, one in particular I remember her son like a lot of people with PMID is non-verbal and she has a small circle of family. During the pandemic, she can speak to her son but her son can’t speak back to her and so she is having no communication at all, that would be something if there was somebody to chat to.* [Male, NGOW, 25-34 years old]

#### Fear of COVID-19

While there was clearly a certain amount of fear among the general population regarding COVID-19, this was exacerbated by the increased vulnerability among those with learning disabilities, as exemplified by the following quotes:

*We were also scared of taking Covid to a family. So, if you did have to meet them you worried about touching anything and all the extra things, you thought what if somebody comes down with it and I’ve been the person that has taken it there.* [Female, NGOW, 55-64 years old]

*Just from myself I know at the very beginning I had a massive fear that my daughter would get sick and have to go into hospital, and I wouldn’t be there. As a parent you spend your entire life interpreting your child for other people and helping them to communicate and be understood themselves. I was in absolute dread that if she got sick and had to go to the hospital, I had visions of myself storming the hospital to try and get into her.* [Female, self-advocate, 35-44 years old]

#### The exhaustion of caring

Exhaustion was a common subtheme expressed by both the self-advocates and NGOW. Family carers were left to cope without the support of day centres, family, friends and with the removal of social care packages. Many faced a disproportionate burden of care leading to increased stress and depression [1, 13].

*Many families had some support but most of them didn’t have the support they really needed and they were already at exhaustion stage prior to the pandemic coming in.* [Female, NGOW, 55-64 years old]

*It’s like when you are in the middle of a crisis you keep going because you have to keep going and then when the crisis starts to subside you start to let out stuff that you have been holding in for a long time. I think we are coming to that phase with staff who are absolutely worn out and fatigued.* [Female, self-advocate, 35-44 years old]

#### “They who shout the loudest”

A recurring theme across the data was how many family carers had to fight for ‘basic’ needs to be met and to have necessary supports provided. There was a feeling among those participants who were self-advocates that they had a black mark against their name. They felt that they had to battle and argue for so long and with such force that service providers were likely to have ‘marked their cards’, meaning that those in positions of authority disapproved of them. One NGO sector participant said:

*They had to fight really hard to have the support put in place and it was all taken away from them* [at the start of the pandemic]. [Female, NGOW, 45-54 years old]

Another member of staff emphasised how necessary it was for family carers to equip themselves with the necessary knowledge to, in a sense, campaign and ‘fight the system’ to have some of their needs met:

*The thing that we have always seen is that when parents are able to kick up a fuss, in the nicest possible way, and are informed with the right information then that is game changing… …You shouldn’t have to feel like you are battling against the system every time you want something done that you are well within your rights to do.* [Male, NGOW, 25-34 years old]

It seemed that family carers who had the ability, strength and capacity to ‘fight the system’ would have their voices heard, and there was a sense that less vocal families might lose out. One self-advocate voiced her anger and the lengths that she had to go to, to have her son’s essential care and support needs met:

*Nobody gives a toss until I start to create a fuss over one thing or another they all assume everything is ok…* …*Even to get him vaccinated I had to create hell*… … *I went around all of my friends and wound them up as well, gave them the same arguments.* [Female, self-advocate, 65+ years old]

I have always got the services that I wanted for [my son], I have always had to shout for them but how awful that you give services to people who bully you into it because that is essentially what I have done over the years. I think that is a corrupt system that lets me do that, but while the system works like that I will work the system to [my son]’s benefit. [Female, self-advocate, 65+ years old]

There was also emphasis that families were not asking for any services above and beyond what they were entitled to.

*So, we were not asking for a Rolls Royce service, we were asking them to just do what they had promised when he got to adulthood. It was a long battle and it got quite horrible at times but anyway we got him in there.* [Female, self-advocate, 65+ years old]

#### “No one arrived to help out” Lack of trust in statutory services

A further theme which emerged from the data was the criticism by some participants regarding statutory services. There was a sense that NGOs were willing to go the extra mile for families, in comparison to statutory services. This is illustrated below where a Female, NGOW, 55–64 years old, described a lack of response from statutory services to continued queries from family carers.

*I’m not trying to blow the [NGO] trumpet but lots of families have said if it hadn’t been for our organisation they don’t know what they would have done or where they would have been.* [Female, NGOW, 45-54 years old]

*I think the third sector organisations have completely held up the community through the pandemic, absolutely and it’s shocking. I was in a meeting a couple of weeks ago with other third sector organisations and yes we don’t have all the red tape and we can respond flexibly but I mean there is no excuse for some of it.* [Female, NGOW, 45-54 years old]

Some participants mentioned the difficulty in communication with, for example, social services, during the pandemic:

*You just can’t imagine how some of the families managed because no one came across the door, no one arrived to help out. They would make phone calls to relevant professionals in social work, GP practices and there was no answer, there was no one willing to come out which was unbelievable for all of us.* [Female, NGOW, 55-64 years old]

A lack of trust was identified between family carers and some statutory services or local authorities. Family carers reported a lack of trust in statutory service providers during the pandemic due to concerns that they were being forgotten, a sentiment echoed by other researchers [35]. Poor communication and ongoing problems with service provision may have further reduced trust and led to family carers turning to NGOs as sources of support and advice:

*The whole redesign of services has meant that families have lost trust in local authorities and services and there wasn’t a lot of trust before. There was never a great partnership because of communication between statutory services, parents and the lack of support. That trust is almost all gone now and how that ever can be brought back I don’t know.* [Female, NGOW, 45-54 years old]

*Fundamentally there is a huge lack of trust between families and local authorities, and local authorities and families. I think local authorities think that families are going to abuse the system which they are not… …A lot of what the people are asking for is not pie in the sky stuff, it’s all relatively low cost realistic stuff that makes sure people are happy and healthy. *[Male, NGOW, 25-34 years old]

The need to consult family carers regarding matters that affect them was identified as an issue, which statutory services neglected to do on a regular basis. This left family carers feeling unheard by statutory services which may have contributed to the erosion of trust. Conversely, NGOs took the time to listen to the concerns of family carers resulting in greater trust and provision of a service which was directly informed by their needs:

*For risk assessments, we were asking families what should be here, what are you thinking? What is an issue for you, what should we be doing to keep you safe? It’s about making families feel included which they don’t feel like through other statutory services. They don’t have that real consultation where they are listened to.* [Female, NGOW, 45-54 years old]

#### Creating an online support programme

One of the main foci of the focus group discussions was encouraging NGOW to think about what a successful support programme for family carers would comprise and might look like. Responses strongly emphasised the need to adopt a coproduction model – i.e. consulting family carers about what *they* would look for in a support programme:

I think anything that you create needs to be created alongside involving experts by experience be that carers themselves, people with learning disabilities, they will come up with the best suggestions of what they think works and what doesn’t. [Female, NGOW, 45-54 years old]

*I suppose we take it so much for granted but the word co-produce, I would say speak to families and ask them what they need, what would work for them, what time of day, what sort of venues, what sort of things would be useful. Some things are so basic but sometimes people develop all these things and they have never actually spoken to somebody about what would work, so that would be my thought.* [Female, NGOW, 55-64 years old]

A common topic discussed was the need for peer support, the extent to which peers have supported each other during the pandemic, and the potential for a support programme to connect people. It was felt that peer support for family carers would reduce feelings of isolation through sharing experiences and creating social connections to support emotional well-being. Family carers have expressed appreciation for support received through the use of technology (videoconferencing) which linked them with family, friends and certain services [20].

*First thing that comes to mind if some sort of reduction in isolation. I think a lot of our families are very lonely so anything that can help that. *[Female, NGOW, 45-54 years old]

*One thing I’ve always thought would be really good and I think it would be difficult to get up and running and also to facilitate it but some sort of buddy system to introduce families to others.* [Male, NGOW, 25-34 years old]

*What people need is a network that they feel they can tap into when they need to. I am a big believer and I think it has helped a lot of people together, somewhere where people can just get together, have a natter and have a coffee. Those circles of support have been lifelines for a lot of people I think during the pandemic. For me if there was something that could really highlight stuff it’s people really seeing the value in those, and they are not just nice to have things, they are actually critical. *[Male, NGOW, 25-34 years old]

Some participants discussed efforts that their organisations made, or that they had been involved in, during the pandemic, which were helpful in supporting family carers. Some NGOs had established online activities to engage those they work with, for example online quizzes, bingo, cookery demonstrations, yoga, mindfulness, wellbeing, music therapy, horticulture, exercise classes.

*We were able to develop our virtual activity programme. Every single member of staff pulled into the development of that programme and what we now have and have consistently had pretty much since the first couple of weeks within the pandemic is a rolling programme of music, drama, storytelling, arts and crafts, sporting activities, carer catch-up, information workshops. *[Female, NGOW, 45-54 years old]

Others felt that an online programme might include a one-stop shop repository of relevant, honest, reliable and easy-accessible information.

*I think that information which is needed is where people can get support for a range of things really: care, support, leisure.* [Female, NGOW, 45-54 years old]

*Let’s say when I was thinking about [my son] going into independent living, I would have liked to have been able to type in something like – can people tell me what arguments to use when I am trying to get my head around how I am going to present my case? Because no person ever presents your case for you, you are the one who is going to have to do that.* [Female, self-advocate, 65+ years]

There was emphasis on the business and exhaustion of family carers and the fact that they would only be able to access support if they were relieved of their caring duties or if it was at the right time of day:

*They had no time and they were absolutely exhausted so it is really difficult to say what would support people online because it just depends where that person is at that point.* [Female, NGOW, 45-54 years old]

*I’m getting asked more and more about the counselling and wellbeing side of things because I think families are now very very tired.* [Female, NGOW, 55-64 years old]

## Discussion

This research adds to the body of evidence demonstrating the impact of the COVID-19 pandemic on the mental health and well-being of family carers through survey [2–4]. It extends this work by exploring this impact on family carers of those with PMID, by capturing the experiences of NGOW and self-advocates across the UK and Ireland. Our qualitative approach has added depth to the descriptions of reductions in mental health experienced by family carers. Our first research question sought to explore the challenges faced by family carers of people with PMID during the COVID-19 pandemic from the perspective of NGOW. Participants recounted how family carers experienced feelings of isolation, confusion, fear and exhaustion. These feelings will likely be echoed by many readers of this journal, however, carers of people with PMID were already experiencing significant strain prior to the pandemic [36, 37] which was then exacerbated by restrictions. It is unknown what the long-term impact of the COVID-19 pandemic will be on family carers, however, efforts should be made to provide appropriate, tailored resources and targeted interventions to reduce any impacts. We would suggest this be achieved through co-production with key stakeholders including family carers and NGOs who possess significant lived experience. Co-production has been successfully used in the creation of a computerised cognitive behavioural therapy package for carers of people with dementia, where it was felt to be central to design of the programme [32].

Our findings highlight the ongoing need for family carers to fight for services and supports to which they are entitled. Family carers who have the time, resources and confidence to advocate for their children with disabilities find this struggle difficult [37]. Knowledgeable, self-advocates described how they had been forced to ‘work the system’ to have their son vaccinated during the COVID-19 pandemic. Family carers who lack the confidence, knowledge or time to fight for service provision for their children may not receive the services they are entitled to [38, 39]. Improved, easily accessible information on the entitlements of families to services should be provided to make carers aware of what services are available. GPs, social workers and other professionals who work closely with family carers should better understand their ongoing needs and provide services which meet these needs. These findings should be considered in the design of future service provision, particularly in the context of ameliorating the impact of the COVID-19 pandemic.

Participants identified a lack, or decrease in trust, with statutory services when compared to NGOs. Services during the COVID-19 pandemic were placed under considerable pressure meaning that many supports, initiatives and some health procedures were not provided [40, 41]. Research has consistently reported that families experienced feelings of abandonment by statutory services during the pandemic [16, 20] with NGOs, who had built relationships with families prior to and throughout the pandemic, stepping in to offer support. This lack of provision could be one reason why trust had been damaged. However, while this may have been most acutely felt during the COVID-19 pandemic the feeling that statutory services do not ‘listen to’ families is a common theme prior to the pandemic [39, 42, 43]. Participants described a lack of response to requests for support from statutory services which left families feeling isolated and uncared for. In contrast, NGOs were perceived as a ‘lifeline’ for families who offered support and fostered a sense of community.

In addition to capturing NGOW views on the impact of the COVID-19 pandemic on family carers our second research question sought to understand what aspects of an online support programme might prove useful in reducing such impacts. Participants felt that including the voices and experiences of family carers in any such programme would be crucial as would some sort of mechanism for connecting people. Non-Governmental Organisation Workers described the efforts their organisations had gone to in providing activities and social events online that were delivered at times suitable for carers. Our recent systematic review of the evidence-base showed a lack of consultation or co-production in the development of online interventions for family carers of individuals with intellectual disabilities (REMOVED FOR BLIND REVIEW). This may be due to therapeutic considerations (e.g. training carers to deal with challenging behaviours), however, interventions which take the needs of family carers into consideration when designing such programmes may address a gap in provision and meet current and future unmet needs. Online programmes offer the potential for a low cost, accessible and adaptable approach to supporting family carers which are accessible from home. Researchers have employed co-production in developing an online mindfulness intervention to reduce stress for family carers of people with ID [5]. This research built on the existing intervention by co-producing a peer mentoring component to the existing mindfulness programme [5]. Other researchers have sought to consult with family carers about their preferences and desire for further supports in the Positive Parenting Program which they achieved through focus group interviews [44]. However, online platforms are not without disadvantage which should be taken into consideration in designing any new programme. For example, families may not possess adequate technology or access to a reliable internet connection [45, 46], may experience technical difficulties [47] or may prefer face to face interactions. Our findings suggest that family carers trust NGOs, therefore, it would be advisable to build on established relationships with these organisations, who have provided ongoing support to families, in some cases for many years, when creating online programmes.

### Policy implications

Policy makers and service providers need to respond to and address the long-term consequences of disrupted care during the COVID-19 pandemic. There is an existing deficit in service provision for many families who may struggled to access adequate supports, a challenge that remains to be addressed. It is important to meaningfully include family carers in the design and implementation of future support programmes to ensure they are acceptable and fit for purpose. There is therefore an opportunity for policy makers and service providers to model future changes to existing or new services in consultations with NGOs to ensure the views, experiences and needs of family carers are heard and addressed.

### Strengths and limitations

To our knowledge, this is the first UK and Republic of Ireland wide study to explore the impact of the COVID-19 pandemic on family carers through the lens of NGOW. This is important as NGOs worked closely with family carers during this period and were an invaluable support for many. A further strength of this study is the use of qualitative methods which adds depth to a number of existing surveys. Research efforts in the early stages of the pandemic sought to gather data quickly, in a responsive fashion. While extremely useful, this data failed to capture the complete picture of individuals’ experiences, a gap which this study addresses. The participants included in this study were aged from between 25–34 and 65 + years. This broad age range suggests that our participants may be representative of NGOW in the UK and Ireland. However, it should be noted that 100% of participants were Caucasian which is unlikely to reflect the diversity of NGOW. Participants included NGOW and self-advocates which adds a crucial perspective to study findings. Self-advocates as carers gave a personal account of how the pandemic impacted on their families and the stresses they themselves experienced. Whilst our study was conducted across the UK and Ireland, it is not possible to fully determine the transferability of the findings to other countries. Ultimately, this study explored the experiences of family carers viewed through the lens of NGOW. Therefore, the findings may not fully represent the experiences of family carers more widely. We are now preparing a follow-on paper using further data which will capture the direct experiences of family carers of people with PMID.

## Conclusions

The reduction in services, coupled with ongoing historical problems with accessing supports, has increased the vulnerability of family carers of those with PMID. These families will continue to provide crucial care for their family members across the lifespan, sometimes to the detriment of their own health and well-being. Non-Governmental Organisations have provided ongoing support during the COVID-19 pandemic and are a trusted source of advice and information for carers. They are thus an important contributor to any efforts to design new, or adapt existing, services. Such services may be delivered in person or online with the caveat that family carers should also be involved.

## Data Availability

All anonymised data will be made available via the UK Data Archive.

## Abbreviations

NGO(s): Non-Governmental Organisation(s)
NGOW: Non-Governmental Organisation Worker
PMID: Profound and Multiple Intellectual Disabilities
UK: United Kingdom
COVID-19: Coronavirus disease
