# Correction: Impact of the COVID-19 pandemic on family carers of those with profound and multiple intellectual disabilities: perspectives from UK and Irish Non-Governmental Organisations

**DOI:** 10.1186/s12889-022-14740-2

**Published:** 2022-12-12

**Authors:** M. A. Linden, T. Forbes, M. Brown, L. Marsh, M. Truesdale, E. McCann, S. Todd, N. Hughes

**Affiliations:** 1 School of Nursing and Midwifery, Queen’s University Belfast, 97 Lisburn Road, Belfast, BT9 7BL Northern Ireland; 2 School of Health and Wellbeing, University of Glasgow, Glasgow, Scotland; 3 Division of Nursing at City, University of London, London, UK; 4 School of Care Sciences, University of South Wales, Caerleon, Wales; 5 Department of Sociological Studies, University of Shefeld, Shefeld, England

**Correction: BMC Public Health 22, 2095 (2022)**

**https://doi.org/10.1186/s12889-022-14560-4**

The original publication of this article [1] contained two missing citations and one missing reference. The incorrect and correct information is shown in this correction article. The original article has been updated.

**Incorect**

- The use of online platforms and telephone support services were a lifeline for many, whilst offering both opportunities (e.g. remote access, availability, reduced travel) and challenges (e.g. access to technology, reliability of technology) (REMOVED FOR BLIND REVIEW)
- Our recent systematic review of the evidence-base showed a lack of consultation or co-production in the development of online interventions for family carers of individuals with intellectual disabilities (REMOVED FOR BLIND REVIEW).

**Correct**

- The use of online platforms and telephone support services were a lifeline for many, whilst offering both opportunities (e.g. remote access, availability, reduced travel) and challenges (e.g. access to technology, reliability of technology) [27].
- Our recent systematic review of the evidence-base showed a lack of consultation or co-production in the development of online interventions for family carers of individuals with intellectual disabilities [27].

[27] Forbes, T., Brown, M., Marsh, L., Truesdale, M., McCann, E., Todd, S., Hughes, N., Linden, M.A. Online support programmes for family carers of people with intellectual disabilities: Systematic review of the international evidence-base. Journal of Applied Research in Intellectual Disabilities. (under review).
